# Retinal vascular occlusion in pregnancy: three case reports and a review of the literature

**DOI:** 10.1186/s13256-022-03369-9

**Published:** 2022-04-21

**Authors:** L. Jürgens, R. Yaici, C. M. Schnitzler, A. K. Fleitmann, M. Roth, K. Schröder, R. Guthoff

**Affiliations:** 1 Department of Ophthalmology, University of Duesseldorf, Moorenstr. 5, D-40225 Düsseldorf, Germany; 2 Department of Obstetrics and Gynaecology, University of Duesseldorf, Düsseldorf, Germany

**Keywords:** Retinal vascular occlusion, Pregnancy, OCT angiography, Cilioretinal artery occlusion, Central retinal vein occlusion, Paracentral acute middle maculopathy

## Abstract

**Background:**

Retinal arterial occlusive events in young patients are rare. However, because of physiological multifactorial adaptations during pregnancy, retinal vascular occlusive disease may occur spontaneously. In addition, a patent foramen ovale is a risk factor for an ischemic thromboembolic event. Since fluorescein angiography, a central tool in the evaluation of these occlusions, should be avoided during pregnancy, optical coherence tomography angiography, a novel technique, offers a good opportunity for visualizing vascular perfusion of retinal tissue.

**Case presentation:**

Here we present a case series of three patients (Caucasian, nonsmoker) who visited our clinic owing to acute visual impairment and central scotoma. Using regular optical coherence tomography and optical coherence tomography angiography, retinal vascular occlusions were detected, thus initiating the evaluation of systemic risk factors. We report two patients (30 and 32 years old) who developed cilioretinal artery occlusion but whose etiology differed: one was of thromboembolic origin associated with patent foramen ovale, while the other was caused by hemodynamic blockade secondary to central retinal vein occlusion. In both cases, optical coherence tomography angiography revealed reperfusion of the cilioretinal artery occlusion. However, transient ischemia led to retinal atrophy after a few weeks. In the third patient (32 years old), 8 weeks after onset of scotoma, optical coherence tomography angiography revealed atrophy of the middle layers and impaired perfusion in the deep capillary plexus, and thus a paracentral acute middle maculopathy was diagnosed. All patients regained normal visual acuity and had otherwise uncomplicated pregnancies, and laboratory blood tests did not reveal any defects or alterations.

**Conclusions:**

As shown here, optical coherence tomography angiography enables risk-free imaging of retinal vessel perfusion during pregnancy. Together with regular optical coherence tomography, it allows one to predict functional outcome according to the existing retinal occlusion-related atrophy.

## Background

Retinal arterial occlusive diseases (RAO) in young patients are rare. Only 11.4% of all RAO occur in people under 50 years of age [1]. In pregnancy however, retinal vascular occlusive diseases may arise spontaneously or a preexisting constitution may deteriorate [2]. There are many physiologic adaptions during pregnancy that can induce vascular occlusive events [3]. These include complex cardiovascular [4–7], hormonal [8, 9], hemostaseological [10, 11], and immunological changes [12, 13]. These adaptions not only increase the risk of a retinal vascular occlusive event but also of stroke during pregnancy. Furthermore and independent of pregnancy, patent foramen ovale (PFO)-related stroke is increasingly recognized as an important etiology of ischemic embolic stroke [14, 15]. Guidelines recommend exclusion of PFO and other sources of embolism in the case of RAO [16].

Normally, fluorescein angiography is a central part of diagnosing a retinal vascular occlusion. It has been established that fluorescein dye crosses the placenta into the fetal circulation [17], yet detrimental effects of fluorescein dye on a fetus have not been documented [18]. However, its use should be avoided during pregnancy. Optical coherence tomography angiography (OCTA) is a novel, noninvasive method for visualization of the functional retinal vessels measuring the movement of red blood cells [19] and therefore represents a good method for analyzing the perfusion of retinal vessels in pregnant patients. OCTA has made it for the first time possible to analyze microvascular changes such as in diabetes mellitus and hypertension during pregnancy.

Here we present a case series of three patients who visited our clinic owing to acute visual impairment. RAO were detected using OCT and OCTA, thus initiating the evaluation of systemic risk factors.

Case presentations

### Case 1

**Fig. 1:**
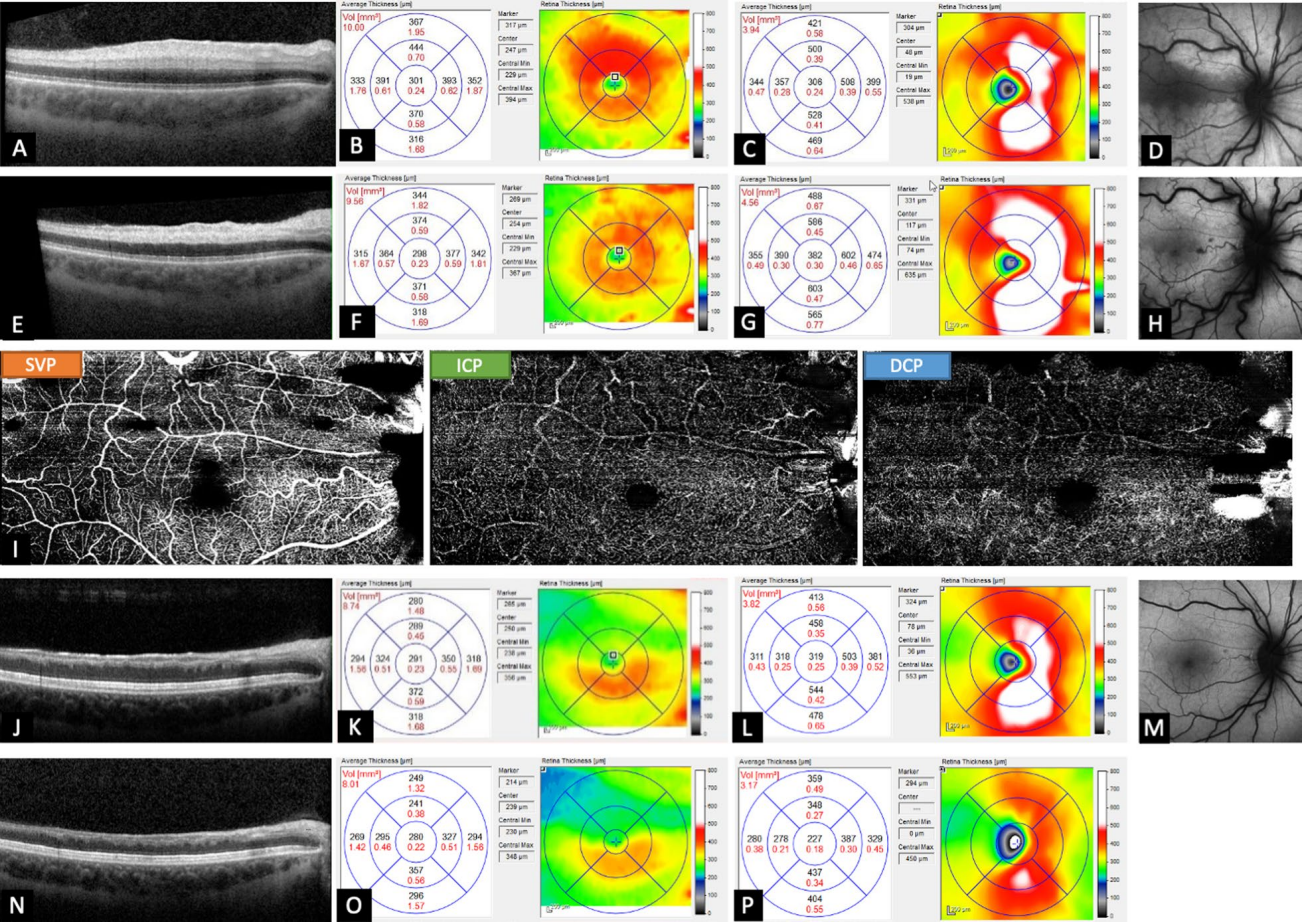
Patient with a reperfused cilioretinal artery occlusion accompanied by a non-ischemic central retinal vein occlusion. Initial optical coherence tomography and fundus autofluorescence showed edema of the inner retinal layers mainly of the superior macular area **A**, **B** and a swollen optic disc (**C**, **D**). After 1 week, macular edema began to decrease (**E**, **F**) while the optic disc edema became more severe (**G**, **H**). Despite difficulties in segmentation, optical coherence tomography angiography demonstrated quite regular perfusion in all three vascular plexuses (superficial vascular, intermediate capillary, and deep capillary plexus) (**I**). After 6 weeks, optical coherence tomography revealed atrophy of the inner retina (**J**, **K**) and decrease of the optic disc edema (**L**, **M**). After 6 months, the inner retina layers were severely atrophic (**N**, **O**) and the superior half of the optic disc showed progressive atrophy (**P**)

A 32-year-old Caucasian pregnant woman (week 19) with a 2-day history of a scotoma of her right eye presented with visual acuity (VA) of 5/200 (Snellen). The ophthalmological history was otherwise unremarkable. Fundoscopy revealed a cilioretinal artery occlusion (CLRAO) accompanied by a non-ischemic central retinal vein occlusion (CRVO). Initial OCT and fundus autofluorescence (Spectralis, Heidelberg Engineering) showed foveal involvement with edema of the inner retinal layers mainly of the superior macular area (Fig. 1A, B) and a swollen optic disc (Fig. 1C, D).

Within 2 days, VA recovered to normal (20/20) and remained stable during the entire follow-up. Within a week, the macular edema began to decrease (Fig. 1E, F) while the optic disc edema became more severe (Fig. 1G, H). The latter corresponded to fundoscopy examination showing a severe optic disc swelling and vascular tortuosities reflecting pre-stasis. OCTA (Spectralis, Heidelberg Engineering) was performed using manufacturer default slab definitions for superficial vascular plexus (SVP; ganglion cell layer—inner plexiform layer), intermediate capillary plexus (ICP; inner plexiform layer—inner nuclear layer), and deep capillary plexus (DCP; inner nuclear layer—outer plexiform layer) as described by Campbell *et al.* [20]. OCTA demonstrated quite regular perfusion in the SVP, ICP, and DCP (Fig. 1I) despite difficulties in segmentation, thus indicating a reperfused CLRAO. The foveal avascular zone (FAZ) was slightly enlarged in the SVP.

At week 3 after the onset of symptoms, there was beginning atrophy of the inner retina resulting in a reduced volume of the superior part of the macula and a persistent, massive increase in optic disc volume. After 6 weeks, the atrophy of the macula progressed, and the optic disc edema began to resolve (Fig. 1J–N). At the last visit, after 6 months, the inner retina layers were severely atrophic and the superior half of the optic disc showed progressive atrophy (Fig. 1O–Q).

The patient was a nonsmoker and had no significant family history of hypercoagulable disorders. Extensive cardiovascular (including long-term electrocardiography and blood pressure measurement, transesophageal echocardiogram, extracranial and transcranial Doppler sonography), hemostaseological testing (protein C/S, antithrombin III, activated protein C resistance, anticardiolipin antibody, anti-β2-microglobulin-antibody, lupus anticoagulant, factor V Leiden mutation, prothrombin G20210A mutation, methylene tetrahydrofolate reductase mutation, platelet function testing, von Willebrand factor antigen and activity), and obstetric examination were unremarkable. Antiplatelet therapy (acetylsalicylic acid 100 mg orally) was initiated.

### Case 2

**Fig. 2:**
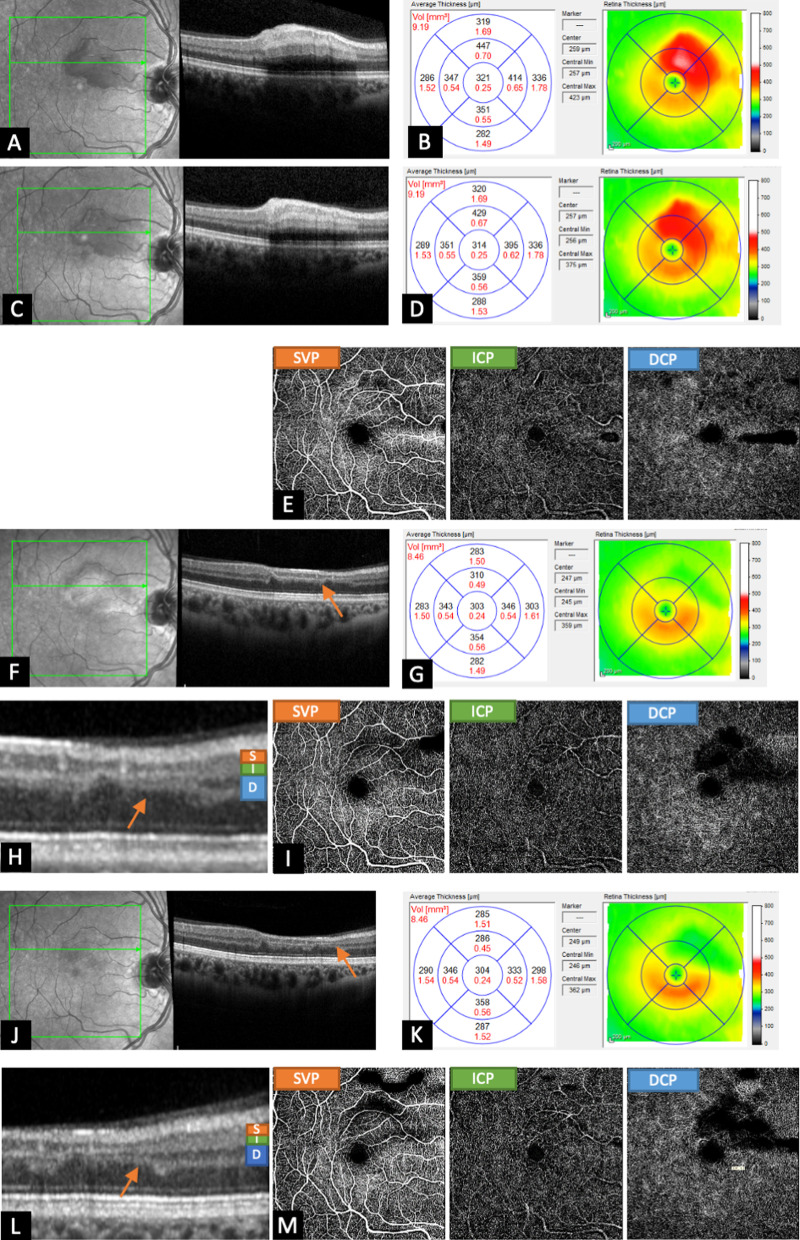
Patient with a reperfused cilioretinal artery-occlusion. Initial optical coherence tomography showed a diffuse swelling of the inner retina layers of the upper macular region (**A**, **B**). Optical coherence tomography at day 4 revealed no increase in edema (**C**, **D**). Optical coherence tomography angiography on day 10 depicts regular vascular structure in all three vascular plexuses (**E**). After 6 weeks, the retinal edema turned into atrophy (**F**, **G**) with thinning of inner nuclear layer and outer plexiform layer (**H**, orange arrows in **F** and **H**). This resulted in reduction of perfusion exclusively in the deep capillary plexus, located in the inner nuclear layer and outer plexiform layer. The superficial vascular plexus and intermediate capillary plexus were not affected (**I**). While there was progression of the retinal atrophy after 6 months (**J**–**L**), especially of the inner nuclear layer and outer plexiform layer (orange arrows in **J** and **L**, there was no progression of the perfusion defects on optical coherence tomography angiography (**M**)

A 30-year-old Caucasian pregnant woman (week 10) presented with a 1-day history of scotoma on the right eye. Fundoscopy and OCT showed a diffuse swelling of the inner retina layers of the upper macular region (Fig. 2A, B). VA was 20/20. The macula edema remained stable until day 4 (Fig. 2C, D). On day 10, OCTA was performed. The three plexuses (SVP, ICP, and DCP) showed regular vascular structure (Fig. 2E), indicating a reperfused CLRAO. Six weeks later, the retinal edema turned into atrophy with a thinning of inner retinal layers, in particular of OPL and INL (Fig. 2F–H, orange arrow pointing to OPL and INL). Only DCP, located in the INL and OPL, showed low perfusion in the OCTA, while the two others vascular plexuses appeared normal. FAZ was not enlarged and intact (Fig. 2I). Therefore, VA was not affected (20/20). At month 6, OCT revealed, compared with week 7, a progressive atrophy of the inner retina (Fig. 2J–M). As seen in week 7, perfusion was impaired in DCP only (Fig. 2N).

The patient was a nonsmoker and had no significant family history of hypercoagulable disorders. Cardiovascular evaluation, as described above, revealed a PFO. Hemostaseological evaluation, as described above, and obstetric examination were unremarkable. Antiplatelet therapy (acetylsalicylic acid 100 mg orally) was initiated, and the patient was recommended to have an interventional PFO closure after pregnancy.

### Case 3

**Fig. 3:**
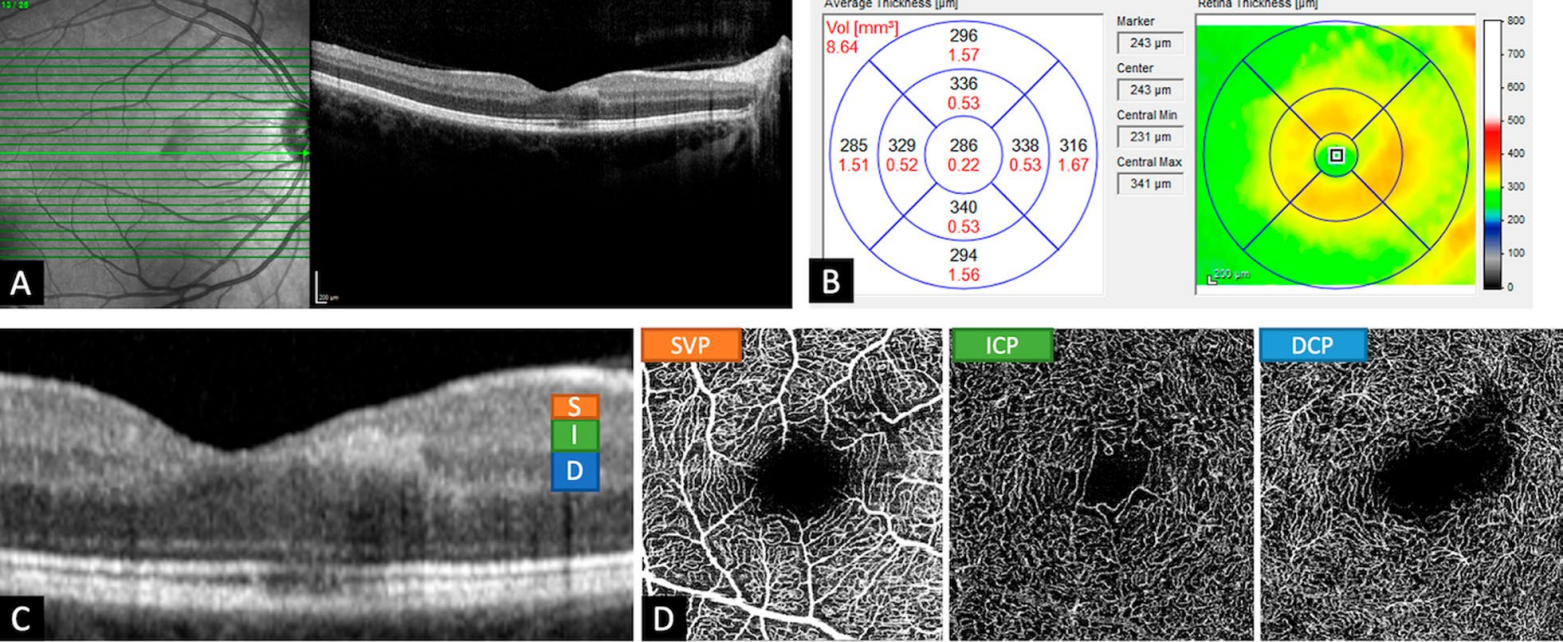
Patient with paracentral acute middle maculopathy. Six weeks after onset of scotoma, infrared image showed a typical defect nasally in the fovea (**A**, **B**). On optical coherence tomography, atrophy of the outer plexiform and inner nuclear layer can be seen (**C**). Optical coherence tomography angiography revealed an irregular vascular network in the intermediate capillary plexus and a nonperfusion area in the deep capillary plexus (**D**)

A 32-year-old Caucasian pregnant patient (week 16) presented with a VA of 20/20 of the right eye. Eight weeks before, she had noticed a central scotoma. She was diagnosed with a paracentral acute middle maculopathy (PAMM). Infrared imaging showed typical defect nasally in the fovea (Fig. 3A). INL and OPL atrophy were revealed on OCT (Fig. 3C) as well as irregular vascular network in ICP and nonperfusion in DCP on OCTA (Fig. 3D).

The patient was a nonsmoker and had no significant family history of hypercoagulable disorders. Cardiovascular risk factor evaluation, as described above, revealed a PFO. Hemostaseological findings, as described above, and obstetric examination were normal. Human immunodeficiency virus (HIV), a possible cause of PAMM, had been ruled out at the beginning of the pregnancy and was not retested. Antiplatelet therapy (acetylsalicylic acid 100 mg orally) was initiated. PFO closure was not recommended by cardiologists.

## Discussion

We present three cases with three distinctly different types of retinal vascular occlusion in pregnancy. Even though case 1 and 2 both depict CLRAO, there is a difference in the pathogenesis. Etiologically, CLRAO is of three distinct types: CLRAO associated with CRVO or hemi-CRVO (like in case 1), non-arteritic CLRAO alone (like in case 2), and arteritic CLRAO associated with giant cell arteritis [21].

The pathogenesis of CLRAO in CRVO is due to transient hemodynamic blockage of the cilioretinal artery caused by a sudden sharp rise in intraluminal pressure in the retinal capillary bed (due to CRVO) above the level of that in the cilioretinal artery. Unlike regular non-arteritic CLRAO, there is no thrombotic or embolic occlusion of the artery in this type [22]. Consistent with this, OCTA in our case showed reperfusion of the cilioretinal artery. (Fig. 1I). The resulting retinal atrophy (Fig. 1L, P) is likely to be caused by transient ischemia lasting longer than 1–2 hours [23, 24].

Non-arteritic CLRAO, as seen in patient 2, is caused by classical thromboembolic occlusion. In our case, the OCTA showed reperfusion 10 days after the onset of the scotoma (Fig. 2E), indicating transient occlusion of the cilioretinal artery. Over time, there was progressive atrophy of the retina, in particular of OPL and INL (Fig. 2H, M). Consistent with this, OCTA depicted no perfusion in the DCP (located in the INL and OPL) in the area of atrophy, while the ICP and SVP showed regular perfusion (Fig. 2I, N).

Paracentral acute middle maculopathy (like in case 3) was first described in 2013 by Sarraf *et al.* [25] and was identified as a variant of acute macular neuroretinopathy. However, it is currently regarded as a different entity from the latter and is a spectral-domain OCT finding characterized by a hyper-reflective band spanning the INL, which typically evolves to INL atrophy in later stages [26] as depicted in Fig. 3C. As previously described by Chu *et al.* [27], we were able to locate an irregular vascular network in ICP and nonperfusion in DCP on OCTA (Fig. 3D)—corresponding to the atrophy in INL. PAMM can be caused by potential infectious, inflammatory, vascular, toxic, and iatrogenic causes [26, 28, 29]. Recently, cases of PAMM during pregnancy were described [30–32]. Like our patient, the three women were described as healthy and had an otherwise uncomplicated pregnancy. One was in her first and two were in their second trimester of pregnancy, like our patient.

OCTA was successfully performed in all three cases. Reperfusion was seen in cases 1 and 2, which could be considered a surrogate marker for better functional outcome in terms of a smaller scotoma due to a smaller atrophic area.

In pregnancy, preexisting conditions may deteriorate owing to hormonal, hematologic, metabolic, cardiovascular, and immunologic changes that induce retinal vascular occlusive events [2, 3]. Associated risk factors for events of retinal vascular occlusion are primary antiphospholipid antibody syndrome, high factor VIII, and low protein S [33]. Also, factor V Leiden mutation has been reported in a case of bilateral retinal vein occlusions [3]. Combined CRVO and CLRAO can occur with increased D-dimer level [34]. However, physiologic changes during pregnancy may play a role. Hormonal alterations increase angiopoietic factors, such as progesterone [35]. Cardiac output and heart rate are physiologically increased [5]. Physiologic hypercoagulability and thrombophilia [11] may also play a role in the pathogenesis of retinal vessel occlusion.

Our patients all had otherwise uncomplicated pregnancies, and laboratory blood tests did not reveal any defects or alterations. Two of the three women (case 2 and 3) possessed a PFO. This is a congenital heart defects with a short-circuit connection between the right and left atrium and is found in around 30% of all healthy adults [36]. Though the majority of patients with PFO are asymptomatic and do not possess an increased risk for developing a stroke, it is likely that other risk factors (such as hypercoagulability) need to be present for a stroke to occur [37]. It was found that PFO-related strokes occur mainly during the first two trimesters [14]. In cases 2 and 3, who both presented PFO, the occlusive event occurred during this time. Post-ischemic management of PFO consists of secondary prophylaxis (antiplatelet therapy) and closure in large-sized PFO shunts [38]. The treatment for retinal vascular occlusions is empirical, and should be performed in close cooperation with hemostaseologists, cardiologists, and obstetricians. We treated all cases with 100 mg acetylsalicylic acid orally, and patient 2 was recommended by cardiologists to undergo interventional PFO closure after pregnancy. Unlike the other two cases, the pathogenesis in case 1, that is, CRVO associated with CLRAO, is not thrombotic or embolic (22). This also reflects the fact that the internal examination was unremarkable. Nocturnal hypotension, being a risk factor, was ruled out. Antiplatelet therapy as secondary prophylaxis was initiated.

## Conclusion

OCTA is a valuable option to noninvasively diagnose and analyze the retinal perfusion status in pregnancy when fundus fluorescein angiography should be avoided. Together with OCT imaging, OCTA allowed us to predict functional outcome in addition to the existing retinal occlusion-related atrophy.

## Data Availability

The data and materials/figures used in the current study are available from the corresponding author on reasonable request.

## Abbreviations

PFO: Patent foramen ovale
OCT: Optical coherence tomography
OCTA: Optical coherence tomography angiography
FAF: Fundus autofluorescence
VA: Visual acuity
CLRAO: Cilioretinal artery occlusion
CRVO: Central retinal vein occlusion
SVP: Superficial vascular plexus
ICP: Intermediate capillary plexus
DCP: Deep capillary plexus
FAZ: Foveal avascular zone
OPL: Outer plexiform layer
INL: Inner nuclear layer
PAMM: Paracentral acute middle maculopathy
RAO: Retinal arterial occlusive disease
